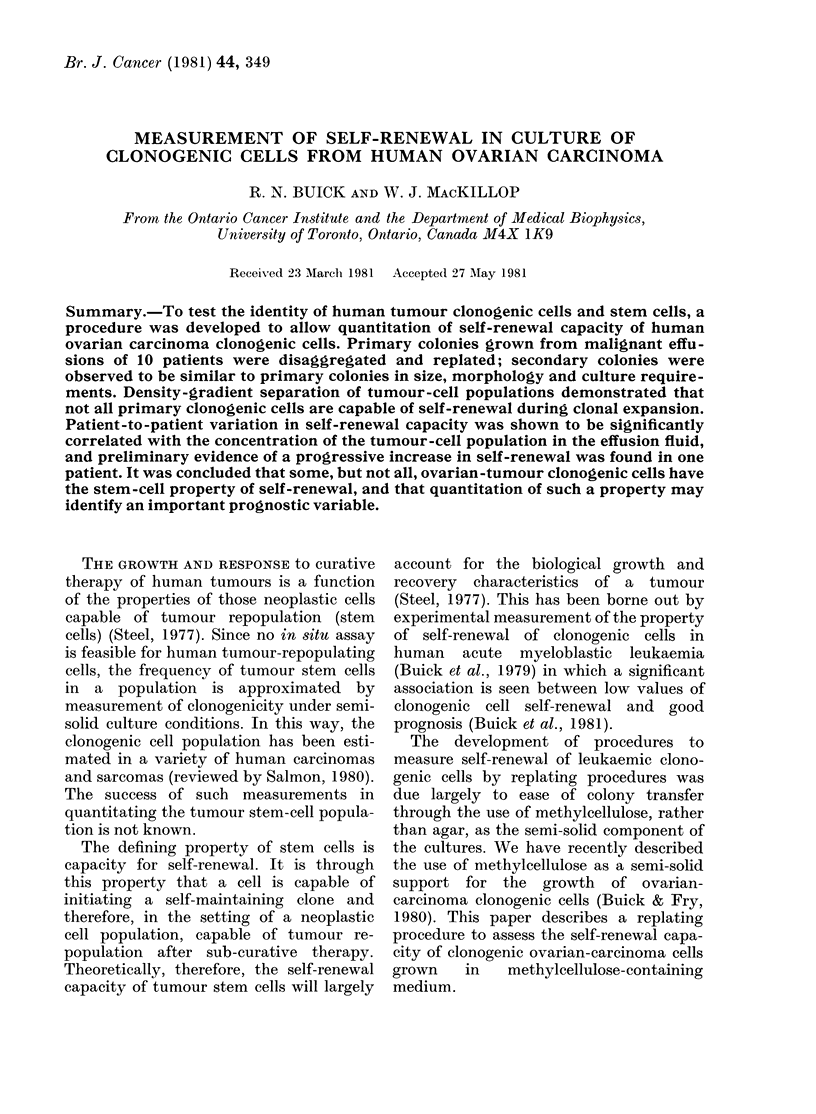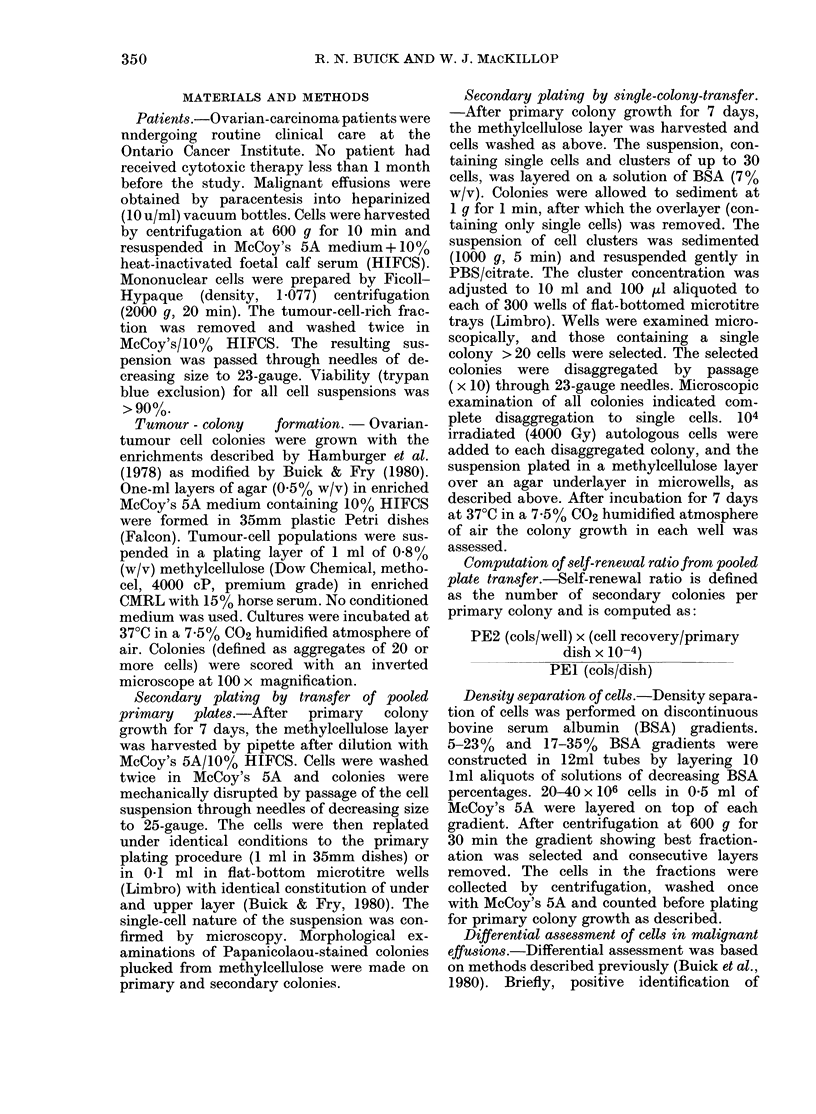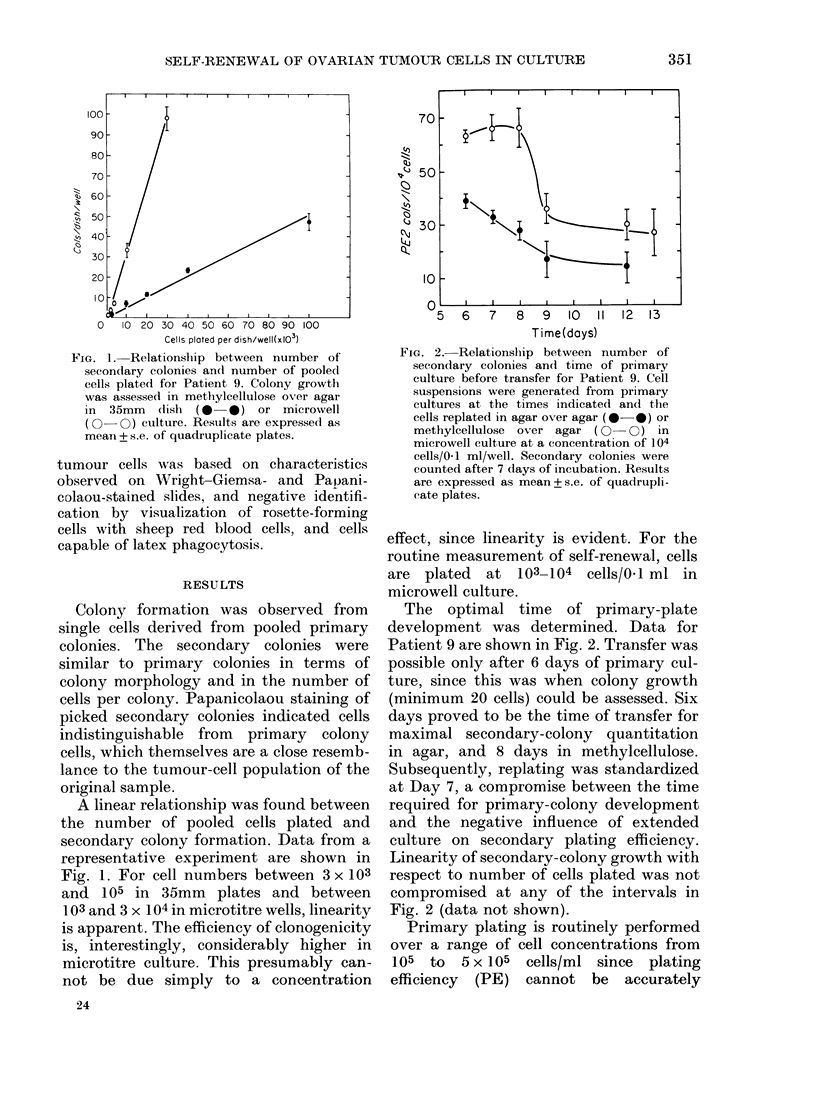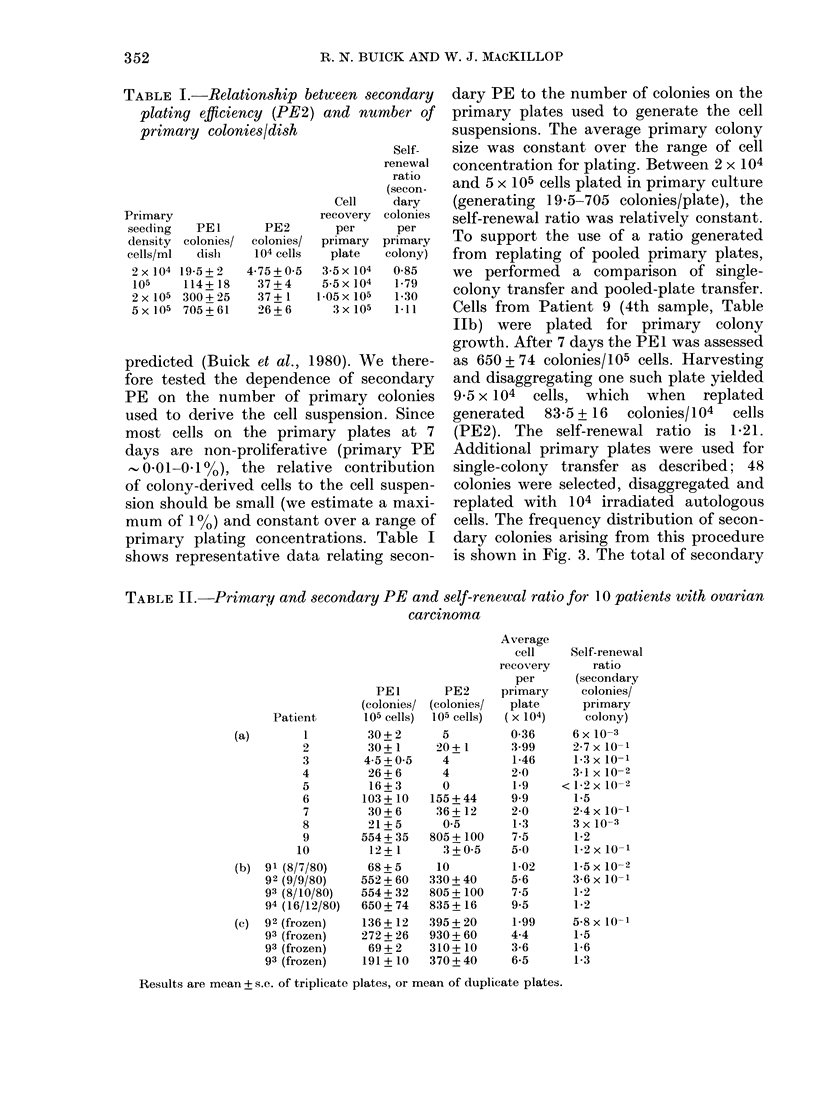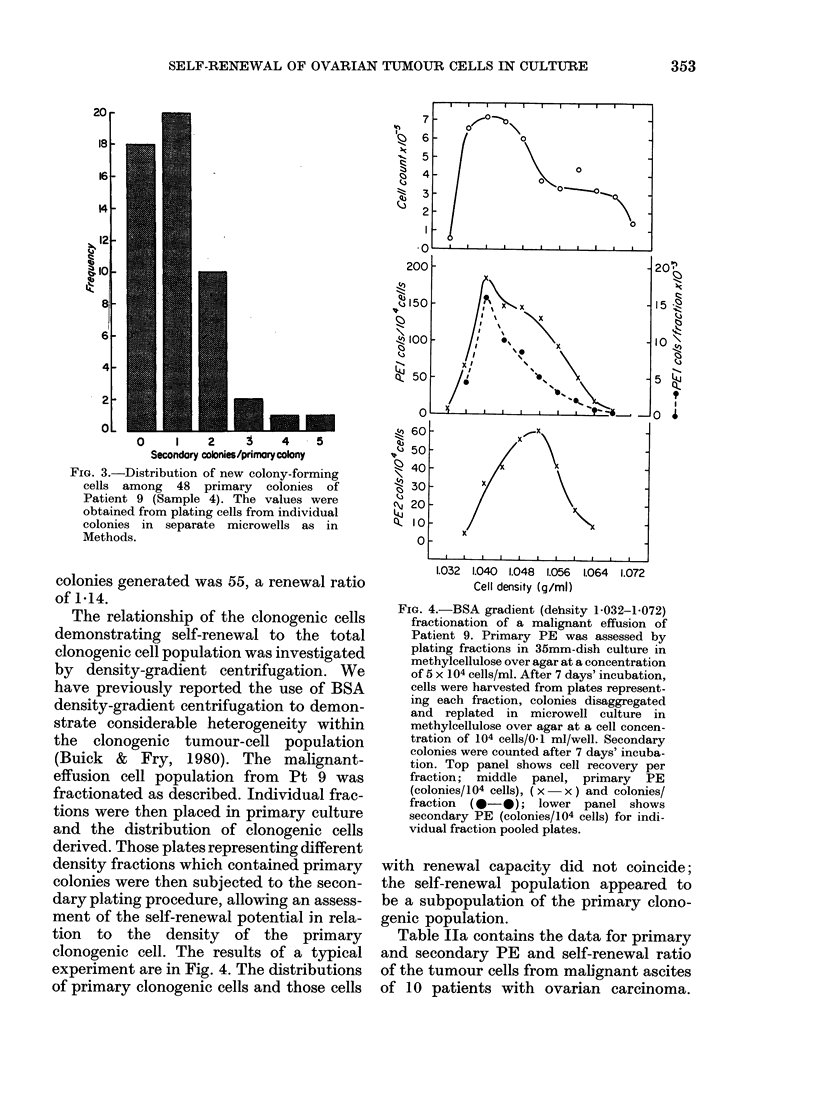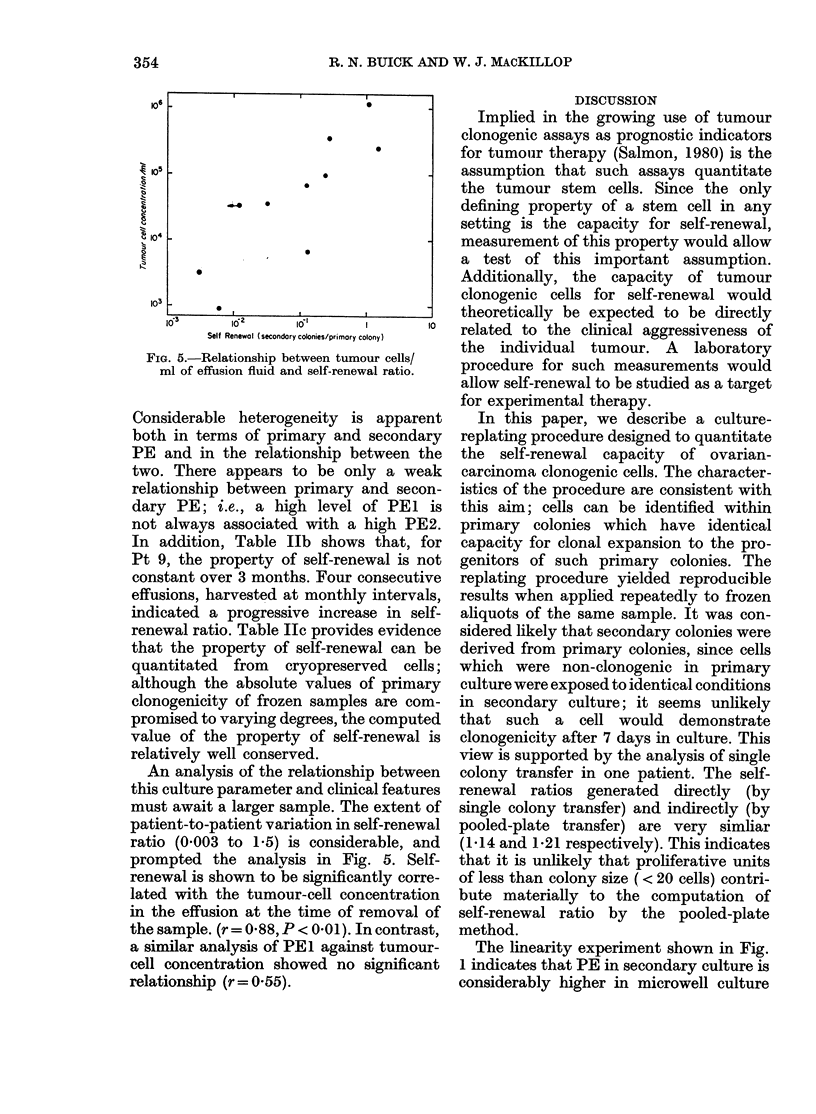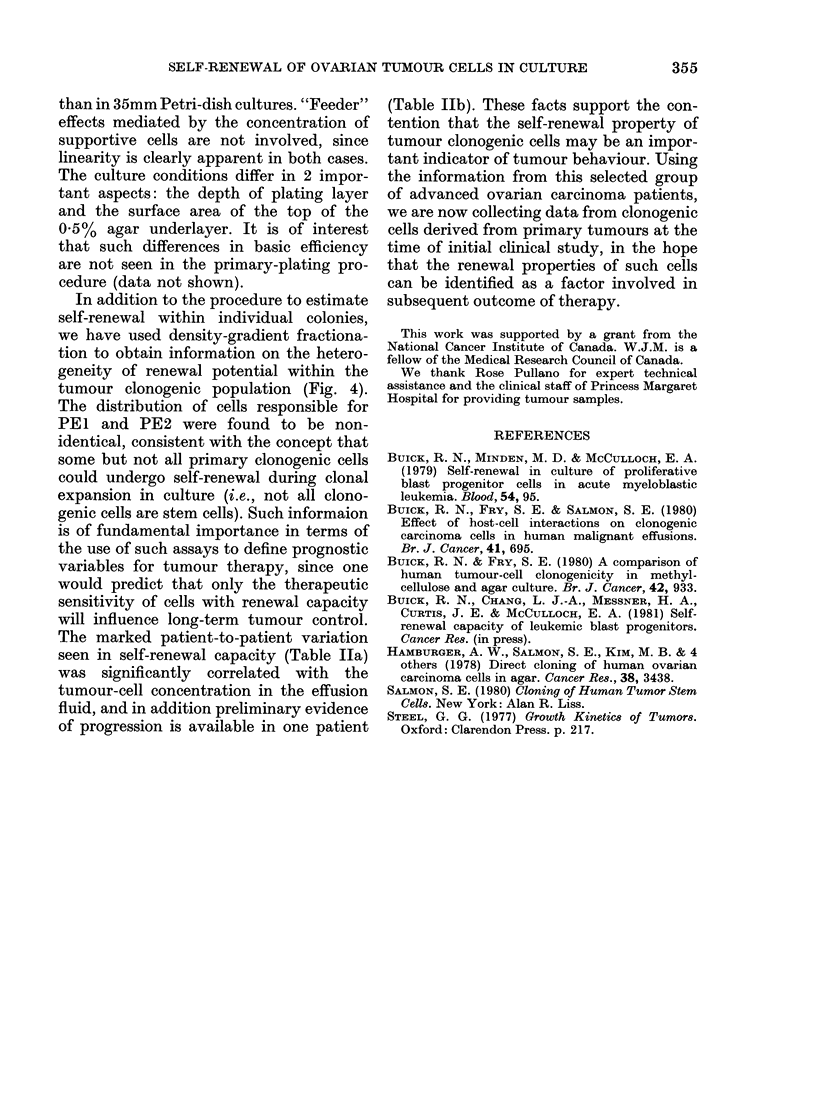# Measurement of self-renewal in culture of clonogenic cells from human ovarian carcinoma.

**DOI:** 10.1038/bjc.1981.191

**Published:** 1981-09

**Authors:** R. N. Buick, W. J. MacKillop

## Abstract

To test the identity of human tumour clonogenic cells and stem cells, a procedure was developed to allow quantitation of self-renewal capacity of human ovarian carcinoma clonogenic cells. Primary colonies grown from malignant effusions of 10 patients were disaggregated and replated; secondary colonies were observed to be similar to primary colonies in size, morphology and culture requirements. Density-gradient separation of tumour-cell populations demonstrated that not all primary clonogenic cells are capable of self-renewal during clonal expansion. Patient-to-patient variation in self-renewal capacity was shown to be significantly correlated with the concentration of the tumour-cell population in the effusion fluid, and preliminary evidence of a progressive increase in self-renewal was found in one patient. It was concluded that some, but not all, ovarian-tumour clonogenic cells have the stem-cell property of self-renewal, and that quantitation of such a property may identify an important prognostic variable.


*Br. J. Cancer* (1981) **44**, 349

### MEASUREMENT OF SELF-RENEWAL IN CULTURE OF CLONOGENIC CELLS FROM HUMAN OVARIAN CARCINOMA

R. N. BUICK AND W. J. MACKILLOP

*From the Ontario Cancer Institute and the Department of Medical Biophysics, University of Toronto, Ontario, Canada M4X 1K9*

Received 23 March 1981 Accepted 27 May 1981

**Summary.—To test the identity of human tumour clonogenic cells and stem cells, a procedure was developed to allow quantitation of self-renewal capacity of human ovarian carcinoma clonogenic cells. Primary colonies grown from malignant effusions of 10 patients were disaggregated and replated; secondary colonies were observed to be similar to primary colonies in size, morphology and culture requirements. Density-gradient separation of tumour-cell populations demonstrated that not all primary clonogenic cells are capable of self-renewal during clonal expansion. Patient-to-patient variation in self-renewal capacity was shown to be significantly correlated with the concentration of the tumour-cell population in the effusion fluid, and preliminary evidence of a progressive increase in self-renewal was found in one patient. It was concluded that some, but not all, ovarian-tumour clonogenic cells have the stem-cell property of self-renewal, and that quantitation of such a property may identify an important prognostic variable.**

THE GROWTH AND RESPONSE to curative therapy of human tumours is a function of the properties of those neoplastic cells capable of tumour repopulation (stem cells) (Steel, 1977). Since no *in situ* assay is feasible for human tumour-repopulating cells, the frequency of tumour stem cells in a population is approximated by measurement of clonogenicity under semi-solid culture conditions. In this way, the clonogenic cell population has been estimated in a variety of human carcinomas and sarcomas (reviewed by Salmon, 1980). The success of such measurements in quantitating the tumour stem-cell population is not known.

The defining property of stem cells is capacity for self-renewal. It is through this property that a cell is capable of initiating a self-maintaining clone and therefore, in the setting of a neoplastic cell population, capable of tumour repopulation after sub-curative therapy. Theoretically, therefore, the self-renewal capacity of tumour stem cells will largely account for the biological growth and recovery characteristics of a tumour (Steel, 1977). This has been borne out by experimental measurement of the property of self-renewal of clonogenic cells in human acute myeloblastic leukaemia (Buick *et al.*, 1979) in which a significant association is seen between low values of clonogenic cell self-renewal and good prognosis (Buick *et al.*, 1981).

The development of procedures to measure self-renewal of leukaemic clonogenic cells by replating procedures was due largely to ease of colony transfer through the use of methylcellulose, rather than agar, as the semi-solid component of the cultures. We have recently described the use of methylcellulose as a semi-solid support for the growth of ovarian-carcinoma clonogenic cells (Buick & Fry, 1980). This paper describes a replating procedure to assess the self-renewal capacity of clonogenic ovarian-carcinoma cells grown in methylcellulose-containing medium.

### MATERIALS AND METHODS

*Patients.*—Ovarian-carcinoma patients were nndergoing routine clinical care at the Ontario Cancer Institute. No patient had received cytotoxic therapy less than 1 month before the study. Malignant effusions were obtained by paracentesis into heparinized (10 u/ml) vacuum bottles. Cells were harvested by centrifugation at 600 *g* for 10 min and resuspended in McCoy's 5A medium + 10% heat-inactivated foetal calf serum (HIFCS). Mononuclear cells were prepared by Ficoll–Hypaque (density, 1·077) centrifugation (2000 *g*, 20 min). The tumour-cell-rich fraction was removed and washed twice in McCoy's/10% HIFCS. The resulting suspension was passed through needles of decreasing size to 23-gauge. Viability (trypan blue exclusion) for all cell suspensions was >90%.

*Tumour - colony formation.* — Ovarian-tumour cell colonies were grown with the enrichments described by Hamburger *et al.* (1978) as modified by Buick & Fry (1980). One-ml layers of agar (0·5% w/v) in enriched McCoy's 5A medium containing 10% HIFCS were formed in 35mm plastic Petri dishes (Falcon). Tumour-cell populations were suspended in a plating layer of 1 ml of 0·8% (w/v) methylcellulose (Dow Chemical, methocel, 4000 cP, premium grade) in enriched CMRL with 15% horse serum. No conditioned medium was used. Cultures were incubated at 37°C in a 7·5% $CO_2$ humidified atmosphere of air. Colonies (defined as aggregates of 20 or more cells) were scored with an inverted microscope at 100× magnification.

*Secondary plating by transfer of pooled primary plates.*—After primary colony growth for 7 days, the methylcellulose layer was harvested by pipette after dilution with McCoy's 5A/10% HIFCS. Cells were washed twice in McCoy's 5A and colonies were mechanically disrupted by passage of the cell suspension through needles of decreasing size to 25-gauge. The cells were then replated under identical conditions to the primary plating procedure (1 ml in 35mm dishes) or in 0·1 ml in flat-bottom microtitre wells (Limbro) with identical constitution of under and upper layer (Buick & Fry, 1980). The single-cell nature of the suspension was confirmed by microscopy. Morphological examinations of Papanicolaou-stained colonies plucked from methylcellulose were made on primary and secondary colonies.

*Secondary plating by single-colony-transfer.*—After primary colony growth for 7 days, the methylcellulose layer was harvested and cells washed as above. The suspension, containing single cells and clusters of up to 30 cells, was layered on a solution of BSA (7% w/v). Colonies were allowed to sediment at 1 *g* for 1 min, after which the overlayer (containing only single cells) was removed. The suspension of cell clusters was sedimented (1000 *g*, 5 min) and resuspended gently in PBS/citrate. The cluster concentration was adjusted to 10 ml and 100 μl aliquoted to each of 300 wells of flat-bottomed microtitre trays (Limbro). Wells were examined microscopically, and those containing a single colony >20 cells were selected. The selected colonies were disaggregated by passage (×10) through 23-gauge needles. Microscopic examination of all colonies indicated complete disaggregation to single cells. $10^4$ irradiated (4000 Gy) autologous cells were added to each disaggregated colony, and the suspension plated in a methylcellulose layer over an agar underlayer in microwells, as described above. After incubation for 7 days at 37°C in a 7·5% $CO_2$ humidified atmosphere of air the colony growth in each well was assessed.

*Computation of self-renewal ratio from pooled plate transfer.*—Self-renewal ratio is defined as the number of secondary colonies per primary colony and is computed as:

$$\frac{\text{PE2 (cols/well)} \times (\text{cell recovery/primary dish} \times 10^{-4})}{\text{PE1 (cols/dish)}}$$

*Density separation of cells.*—Density separation of cells was performed on discontinuous bovine serum albumin (BSA) gradients. 5–23% and 17–35% BSA gradients were constructed in 12ml tubes by layering 10 1ml aliquots of solutions of decreasing BSA percentages. 20–40 × $10^6$ cells in 0·5 ml of McCoy's 5A were layered on top of each gradient. After centrifugation at 600 *g* for 30 min the gradient showing best fractionation was selected and consecutive layers removed. The cells in the fractions were collected by centrifugation, washed once with McCoy's 5A and counted before plating for primary colony growth as described.

*Differential assessment of cells in malignant effusions.*—Differential assessment was based on methods described previously (Buick *et al.*, 1980). Briefly, positive identification of

FIG. 1.—Relationship between number of secondary colonies and number of pooled cells plated for Patient 9. Colony growth was assessed in methylcellulose over agar in 35mm dish (●—●) or microwell (○—○) culture. Results are expressed as mean ± s.e. of quadruplicate plates.

FIG. 2.—Relationship between number of secondary colonies and time of primary culture before transfer for Patient 9. Cell suspensions were generated from primary cultures at the times indicated and the cells replated in agar over agar (●—●) or methylcellulose over agar (○—○) in microwell culture at a concentration of $10^4$ cells/0·1 ml/well. Secondary colonies were counted after 7 days of incubation. Results are expressed as mean ± s.e. of quadruplicate plates.

tumour cells was based on characteristics observed on Wright–Giemsa- and Papanicolaou-stained slides, and negative identification by visualization of rosette-forming cells with sheep red blood cells, and cells capable of latex phagocytosis.

### RESULTS

Colony formation was observed from single cells derived from pooled primary colonies. The secondary colonies were similar to primary colonies in terms of colony morphology and in the number of cells per colony. Papanicolaou staining of picked secondary colonies indicated cells indistinguishable from primary colony cells, which themselves are a close resemblance to the tumour-cell population of the original sample.

A linear relationship was found between the number of pooled cells plated and secondary colony formation. Data from a representative experiment are shown in Fig. 1. For cell numbers between $3 \times 10^3$ and $10^5$ in 35mm plates and between $10^3$ and $3 \times 10^4$ in microtitre wells, linearity is apparent. The efficiency of clonogenicity is, interestingly, considerably higher in microtitre culture. This presumably cannot be due simply to a concentration effect, since linearity is evident. For the routine measurement of self-renewal, cells are plated at $10^3$–$10^4$ cells/0·1 ml in microwell culture.

The optimal time of primary-plate development was determined. Data for Patient 9 are shown in Fig. 2. Transfer was possible only after 6 days of primary culture, since this was when colony growth (minimum 20 cells) could be assessed. Six days proved to be the time of transfer for maximal secondary-colony quantitation in agar, and 8 days in methylcellulose. Subsequently, replating was standardized at Day 7, a compromise between the time required for primary-colony development and the negative influence of extended culture on secondary plating efficiency. Linearity of secondary-colony growth with respect to number of cells plated was not compromised at any of the intervals in Fig. 2 (data not shown).

Primary plating is routinely performed over a range of cell concentrations from $10^5$ to $5 \times 10^5$ cells/ml since plating efficiency (PE) cannot be accurately

TABLE I.—*Relationship between secondary plating efficiency (PE2) and number of primary colonies/dish*

| Primary seeding density cells/ml | PE1 colonies/ dish | PE2 colonies/ $10^4$ cells | Cell recovery per primary plate | Self-renewal ratio (secondary colonies per primary colony) |
|---|---|---|---|---|
| $2\times10^4$ | $19{\cdot}5\pm2$ | $4{\cdot}75\pm0{\cdot}5$ | $3{\cdot}5\times10^4$ | 0·85 |
| $10^5$ | $114\pm18$ | $37\pm4$ | $5{\cdot}5\times10^4$ | 1·79 |
| $2\times10^5$ | $300\pm25$ | $37\pm1$ | $1{\cdot}05\times10^5$ | 1·30 |
| $5\times10^5$ | $705\pm61$ | $26\pm6$ | $3\times10^5$ | 1·11 |

predicted (Buick *et al.*, 1980). We therefore tested the dependence of secondary PE on the number of primary colonies used to derive the cell suspension. Since most cells on the primary plates at 7 days are non-proliferative (primary PE $\sim$0·01–0·1%), the relative contribution of colony-derived cells to the cell suspension should be small (we estimate a maximum of 1%) and constant over a range of primary plating concentrations. Table I shows representative data relating secondary PE to the number of colonies on the primary plates used to generate the cell suspensions. The average primary colony size was constant over the range of cell concentration for plating. Between $2\times10^4$ and $5\times10^5$ cells plated in primary culture (generating 19·5–705 colonies/plate), the self-renewal ratio was relatively constant. To support the use of a ratio generated from replating of pooled primary plates, we performed a comparison of single-colony transfer and pooled-plate transfer. Cells from Patient 9 (4th sample, Table IIb) were plated for primary colony growth. After 7 days the PE1 was assessed as $650\pm74$ colonies/$10^5$ cells. Harvesting and disaggregating one such plate yielded $9{\cdot}5\times10^4$ cells, which when replated generated $83{\cdot}5\pm16$ colonies/$10^4$ cells (PE2). The self-renewal ratio is 1·21. Additional primary plates were used for single-colony transfer as described; 48 colonies were selected, disaggregated and replated with $10^4$ irradiated autologous cells. The frequency distribution of secondary colonies arising from this procedure is shown in Fig. 3. The total of secondary

TABLE II.—*Primary and secondary PE and self-renewal ratio for* 10 *patients with ovarian carcinoma*

| | Patient | PE1 (colonies/ $10^5$ cells) | PE2 (colonies/ $10^5$ cells) | Average cell recovery per primary plate ($\times10^4$) | Self-renewal ratio (secondary colonies/ primary colony) |
|---|---|---|---|---|---|
| (a) | 1 | $30\pm2$ | 5 | 0·36 | $6\times10^{-3}$ |
| | 2 | $30\pm1$ | $20\pm1$ | 3·99 | $2{\cdot}7\times10^{-1}$ |
| | 3 | $4{\cdot}5\pm0{\cdot}5$ | 4 | 1·46 | $1{\cdot}3\times10^{-1}$ |
| | 4 | $26\pm6$ | 4 | 2·0 | $3{\cdot}1\times10^{-2}$ |
| | 5 | $16\pm3$ | 0 | 1·9 | $<1{\cdot}2\times10^{-2}$ |
| | 6 | $103\pm10$ | $155\pm44$ | 9·9 | 1·5 |
| | 7 | $30\pm6$ | $36\pm12$ | 2·0 | $2{\cdot}4\times10^{-1}$ |
| | 8 | $21\pm5$ | 0·5 | 1·3 | $3\times10^{-3}$ |
| | 9 | $554\pm35$ | $805\pm100$ | 7·5 | 1·2 |
| | 10 | $12\pm1$ | $3\pm0{\cdot}5$ | 5·0 | $1{\cdot}2\times10^{-1}$ |
| (b) | $9^1$ (8/7/80) | $68\pm5$ | 10 | 1·02 | $1{\cdot}5\times10^{-2}$ |
| | $9^2$ (9/9/80) | $552\pm60$ | $330\pm40$ | 5·6 | $3{\cdot}6\times10^{-1}$ |
| | $9^3$ (8/10/80) | $554\pm32$ | $805\pm100$ | 7·5 | 1·2 |
| | $9^4$ (16/12/80) | $650\pm74$ | $835\pm16$ | 9·5 | 1·2 |
| (c) | $9^2$ (frozen) | $136\pm12$ | $395\pm20$ | 1·99 | $5{\cdot}8\times10^{-1}$ |
| | $9^3$ (frozen) | $272\pm26$ | $930\pm60$ | 4·4 | 1·5 |
| | $9^3$ (frozen) | $69\pm2$ | $310\pm10$ | 3·6 | 1·6 |
| | $9^3$ (frozen) | $191\pm10$ | $370\pm40$ | 6·5 | 1·3 |

Results are mean $\pm$ s.e. of triplicate plates, or mean of duplicate plates.

FIG. 3.—Distribution of new colony-forming cells among 48 primary colonies of Patient 9 (Sample 4). The values were obtained from plating cells from individual colonies in separate microwells as in Methods.

FIG. 4.—BSA gradient (density 1·032–1·072) fractionation of a malignant effusion of Patient 9. Primary PE was assessed by plating fractions in 35mm-dish culture in methylcellulose over agar at a concentration of 5 x $10^4$ cells/ml. After 7 days' incubation, cells were harvested from plates representing each fraction, colonies disaggregated and replated in microwell culture in methylcellulose over agar at a cell concentration of $10^4$ cells/0·1 ml/well. Secondary colonies were counted after 7 days' incubation. Top panel shows cell recovery per fraction; middle panel, primary PE (colonies/$10^4$ cells), (x — x) and colonies/fraction (●—●); lower panel shows secondary PE (colonies/$10^4$ cells) for individual fraction pooled plates.

colonies generated was 55, a renewal ratio of 1·14.

The relationship of the clonogenic cells demonstrating self-renewal to the total clonogenic cell population was investigated by density-gradient centrifugation. We have previously reported the use of BSA density-gradient centrifugation to demonstrate considerable heterogeneity within the clonogenic tumour-cell population (Buick & Fry, 1980). The malignant-effusion cell population from Pt 9 was fractionated as described. Individual fractions were then placed in primary culture and the distribution of clonogenic cells derived. Those plates representing different density fractions which contained primary colonies were then subjected to the secondary plating procedure, allowing an assessment of the self-renewal potential in relation to the density of the primary clonogenic cell. The results of a typical experiment are in Fig. 4. The distributions of primary clonogenic cells and those cells with renewal capacity did not coincide; the self-renewal population appeared to be a subpopulation of the primary clonogenic population.

Table IIa contains the data for primary and secondary PE and self-renewal ratio of the tumour cells from malignant ascites of 10 patients with ovarian carcinoma.

FIG. 5.—Relationship between tumour cells/ml of effusion fluid and self-renewal ratio.

Considerable heterogeneity is apparent both in terms of primary and secondary PE and in the relationship between the two. There appears to be only a weak relationship between primary and secondary PE; *i.e.*, a high level of PE1 is not always associated with a high PE2. In addition, Table IIb shows that, for Pt 9, the property of self-renewal is not constant over 3 months. Four consecutive effusions, harvested at monthly intervals, indicated a progressive increase in self-renewal ratio. Table IIc provides evidence that the property of self-renewal can be quantitated from cryopreserved cells; although the absolute values of primary clonogenicity of frozen samples are compromised to varying degrees, the computed value of the property of self-renewal is relatively well conserved.

An analysis of the relationship between this culture parameter and clinical features must await a larger sample. The extent of patient-to-patient variation in self-renewal ratio (0·003 to 1·5) is considerable, and prompted the analysis in Fig. 5. Self-renewal is shown to be significantly correlated with the tumour-cell concentration in the effusion at the time of removal of the sample. ($r = 0{\cdot}88, P < 0{\cdot}01$). In contrast, a similar analysis of PE1 against tumour-cell concentration showed no significant relationship ($r = 0{\cdot}55$).

DISCUSSION

Implied in the growing use of tumour clonogenic assays as prognostic indicators for tumour therapy (Salmon, 1980) is the assumption that such assays quantitate the tumour stem cells. Since the only defining property of a stem cell in any setting is the capacity for self-renewal, measurement of this property would allow a test of this important assumption. Additionally, the capacity of tumour clonogenic cells for self-renewal would theoretically be expected to be directly related to the clinical aggressiveness of the individual tumour. A laboratory procedure for such measurements would allow self-renewal to be studied as a target for experimental therapy.

In this paper, we describe a culture-replating procedure designed to quantitate the self-renewal capacity of ovarian-carcinoma clonogenic cells. The characteristics of the procedure are consistent with this aim; cells can be identified within primary colonies which have identical capacity for clonal expansion to the progenitors of such primary colonies. The replating procedure yielded reproducible results when applied repeatedly to frozen aliquots of the same sample. It was considered likely that secondary colonies were derived from primary colonies, since cells which were non-clonogenic in primary culture were exposed to identical conditions in secondary culture; it seems unlikely that such a cell would demonstrate clonogenicity after 7 days in culture. This view is supported by the analysis of single colony transfer in one patient. The self-renewal ratios generated directly (by single colony transfer) and indirectly (by pooled-plate transfer) are very simliar (1·14 and 1·21 respectively). This indicates that it is unlikely that proliferative units of less than colony size (< 20 cells) contribute materially to the computation of self-renewal ratio by the pooled-plate method.

The linearity experiment shown in Fig. 1 indicates that PE in secondary culture is considerably higher in microwell culture than in 35mm Petri-dish cultures. "Feeder" effects mediated by the concentration of supportive cells are not involved, since linearity is clearly apparent in both cases. The culture conditions differ in 2 important aspects: the depth of plating layer and the surface area of the top of the 0·5% agar underlayer. It is of interest that such differences in basic efficiency are not seen in the primary-plating procedure (data not shown).

In addition to the procedure to estimate self-renewal within individual colonies, we have used density-gradient fractionation to obtain information on the heterogeneity of renewal potential within the tumour clonogenic population (Fig. 4). The distribution of cells responsible for PE1 and PE2 were found to be non-identical, consistent with the concept that some but not all primary clonogenic cells could undergo self-renewal during clonal expansion in culture (*i.e.*, not all clonogenic cells are stem cells). Such informaion is of fundamental importance in terms of the use of such assays to define prognostic variables for tumour therapy, since one would predict that only the therapeutic sensitivity of cells with renewal capacity will influence long-term tumour control. The marked patient-to-patient variation seen in self-renewal capacity (Table IIa) was significantly correlated with the tumour-cell concentration in the effusion fluid, and in addition preliminary evidence of progression is available in one patient (Table IIb). These facts support the contention that the self-renewal property of tumour clonogenic cells may be an important indicator of tumour behaviour. Using the information from this selected group of advanced ovarian carcinoma patients, we are now collecting data from clonogenic cells derived from primary tumours at the time of initial clinical study, in the hope that the renewal properties of such cells can be identified as a factor involved in subsequent outcome of therapy.

This work was supported by a grant from the National Cancer Institute of Canada. W.J.M. is a fellow of the Medical Research Council of Canada.

We thank Rose Pullano for expert technical assistance and the clinical staff of Princess Margaret Hospital for providing tumour samples.

### REFERENCES

BUICK, R. N., MINDEN, M. D. & MCCULLOCH, E. A. (1979) Self-renewal in culture of proliferative blast progenitor cells in acute myeloblastic leukemia. *Blood*, **54**, 95.

BUICK, R. N., FRY, S. E. & SALMON, S. E. (1980) Effect of host-cell interactions on clonogenic carcinoma cells in human malignant effusions. *Br. J. Cancer*, **41**, 695.

BUICK, R. N. & FRY, S. E. (1980) A comparison of human tumour-cell clonogenicity in methylcellulose and agar culture. *Br. J. Cancer*, **42**, 933.

BUICK, R. N., CHANG, L. J.-A., MESSNER, H. A., CURTIS, J. E. & MCCULLOCH, E. A. (1981) Self-renewal capacity of leukemic blast progenitors. *Cancer Res.* (in press).

HAMBURGER, A. W., SALMON, S. E., KIM, M. B. & 4 others (1978) Direct cloning of human ovarian carcinoma cells in agar. *Cancer Res.*, **38**, 3438.

SALMON, S. E. (1980) *Cloning of Human Tumor Stem Cells*. New York: Alan R. Liss.

STEEL, G. G. (1977) *Growth Kinetics of Tumors.* Oxford: Clarendon Press. p. 217.